# Alignment-Free and High-Frequency Compensation in Face Hallucination

**DOI:** 10.1155/2014/903160

**Published:** 2014-02-12

**Authors:** Yen-Wei Chen, So Sasatani, Xian-Hua Han

**Affiliations:** ^1^College of Computer Science and Information Technology, Central South University of Forestry and Technology, Hunan 410004, China; ^2^College of Information Science and Engineering, Ritsumeikan University, Shiga 525-8577, Japan

## Abstract

Face hallucination is one of learning-based super resolution techniques, which is focused on resolution enhancement of facial images. Though face hallucination is a powerful and useful technique, some detailed high-frequency components cannot be recovered. It also needs accurate alignment between training samples. In this paper, we propose a high-frequency compensation framework based on residual images for face hallucination method in order to improve the reconstruction performance. The basic idea of proposed framework is to reconstruct or estimate a residual image, which can be used to compensate the high-frequency components of the reconstructed high-resolution image. Three approaches based on our proposed framework are proposed. We also propose a patch-based alignment-free face hallucination. In the patch-based face hallucination, we first segment facial images into overlapping patches and construct training patch pairs. For an input low-resolution (LR) image, the overlapping patches are also used to obtain the corresponding high-resolution (HR) patches by face hallucination. The whole HR image can then be reconstructed by combining all of the HR patches. Experimental results show that the high-resolution images obtained using our proposed approaches can improve the quality of those obtained by conventional face hallucination method even if the training data set is unaligned.

## 1. Introduction

There is a high demand for high-resolution (HR) images such as video surveillance, remote sensing, and medical imaging because high-resolution images can reveal more information than low-resolution images. However, it is hard to improve the image resolution by replacing sensors because of the high cost, hardware physical limits. Super resolution image reconstruction (SR) is one promising technique to solve the problem [1, 2]. SR can be broadly classified into two families of methods: (1) the classical multiframe super resolution [2] and (2) the single-frame super resolution, which is also known as example-based or learning-based super resolution [3–5]. In the classical multiimage SR, the HR image is reconstructed by combining subpixel-aligned multiimages (LR images). In the learning-based SR, the HR image is reconstructed by learning correspondence between low and high-resolution image patches from a database.

Face hallucination is one of learning-based SR techniques proposed by Baker and Kanade [1, 6], which is focused on resolution enhancement of facial images. To date, a lot of algorithms of face hallucination methods have been proposed [7–12]. Though face hallucination is a powerful and useful technique, some detailed high-frequency components cannot be recovered. In this paper, we propose a high-frequency compensation framework based on residual images for face hallucination method in order to improve the reconstruction performance. The basic idea of proposed framework is to reconstruct or estimate a residual image, which can be used to compensate the high-frequency components of the reconstructed high-resolution image. Three approaches based on our proposed framework are proposed. We also propose a patch-based alignment-free face hallucination method. In the patch-based face hallucination, we first segment facial images into overlapping patches and construct training patch pairs. For an input LR image, the overlapping patches are also used to obtain the corresponding HR patches by face hallucination. The whole HR image can then be reconstructed by combining all of the HR patches.

The paper is organized as follows. In Section 2, we describe the conventional face hallucination method. Our proposed residual image compensation methods are presented in Section 3. Our proposed patch-based alignment-free method is presented in Section 4.  Section 5 presents experimental results and quantitative evaluation.  Section 6 summarizes our conclusions.

## 2. Face Hallucination

The face hallucination method is one of learning-based SR methods, which is proposed for resolution enhancement of facial images [6–12]. In this section, we briefly introduce the basic concept of face hallucination, which is shown in Figure 1.

The basic idea of face hallucination is that a face image can be reconstructed from other face images by linear combination because all facial images have a similar structure. In face hallucination, an input LR image can be represented as a linear sum of the LR training images along with some learned coefficients. Due to the correlation between the LR and HR images in the training dataset, the output HR image can also be calculated by finding the linear sum of the corresponding HR images using the same coefficients.

We represent a two-dimensional face image using a column vector of all pixel values, and **X**_*l*_ represents the input LR face image. HR training images are denoted by **H** = [**H**_1_, **H**_2_,…, **H**_*M*_], and the corresponding LR training images are **L** = [**L**_1_, **L**_2_,…, **L**_*M*_], where *M* is the number of training image pairs. First, we interpolate the LR training images and the input (test) LR image to the resolution space of the HR training images, denoted by $\tilde{L}=[{\tilde{L}}_{1},{\tilde{L}}_{2},\ldots ,{\tilde{L}}_{M}]$ and ${\tilde{X}}_{l}$, respectively. ${\tilde{X}}_{l}$ may be represented by a linear sum of interpolated training LR images using

(1) $$\begin{array}{c} {\tilde{X}}_{l}=c_{1}{\tilde{L}}_{1}+c_{2}{\tilde{L}}_{2}+\cdots +c_{M}{\tilde{L}}_{M}, \end{array}$$

where **C** = [*c*_1_, *c*_2_,…, *c*_*M*_] are the weight coefficients, satisfying the following constraint:

(2) $$\begin{array}{c} c_{1}+c_{2}+\cdots +c_{M}=1. \end{array}$$

The optimal weights can be calculated by minimizing the error in reconstructing the input LR image ${\tilde{X}}_{l}$ from training LR images. This error is defined in (3). After substitution of the constraints in (2) into (3), the weight vector may be obtained using covariance matrix **S** in (4). So,

(3) $$\begin{array}{c} \varepsilon \left(C\right)={\left|X_{h}-\tilde{X}\right|}^{2}={\left|X_{h}-\overset{M}{\underset{i=1}{\sum }}c_{i}{\tilde{L}}_{i}\right|}^{2}, \end{array}$$

(4) $$\begin{array}{c} C=\frac{S^{-1}1}{1^{T}S^{-1}1},\;\left(S={\left({\tilde{X}}_{l}1^{T}-\tilde{L}\right)}^{T}\left({\tilde{X}}_{l}1^{T}-\tilde{L}\right)\right). \end{array}$$

After obtaining the coefficients for reconstructing the input LR image with LR training images as given in (1), we replace $\tilde{L}$ with **H** using the same coefficients **C**. Subsequently, the HR image **X**_*h*_ can be obtained using

(5) $$\begin{array}{c} X_{h}=c_{1}H_{1}+c_{2}H_{2}+\cdots +c_{M}H_{M}. \end{array}$$

## 3. High Frequency Compensations Using Residual Images

**Figure 2:**
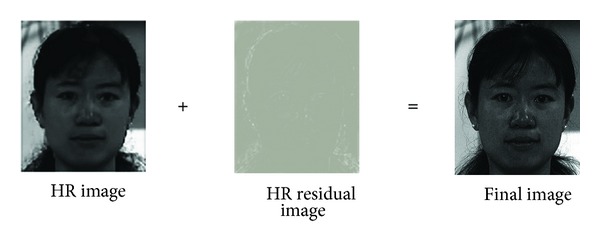
Framework for recovering high-frequency components in face hallucination.

Though face hallucination is a powerful and useful technique, some detailed high-frequency components cannot be recovered. In this paper, we propose a high-frequency compensation framework based on residual images for face hallucination method in order to improve the reconstruction performance. The basic idea of proposed framework is to reconstruct or estimate a residual image, which can be used to compensate the high-frequency components of the reconstructed high-resolution image as shown in Figure 2.

**Figure 3:**
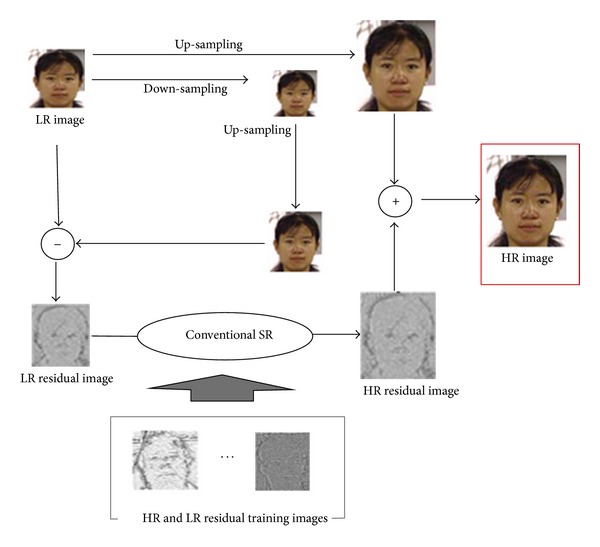
Proposed super resolution method 1.

In order to estimate the residual image, we construct a new residual image database (pairs of LR and HR residual images) in addition to existing image database (pairs of LR and HR images) for training. The construction of the residual image database is shown in Figure 3. The HR and LR training image pairs [**H**_1_, **H**_2_,…, **H**_*M*_] and [**L**_1_, **L**_2_,…, **L**_*M*_] already exist in conventional face hallucination. With conventional face hallucination, for each LR training image **L**, the other *M* − 1 training image pairs are used to obtain the approximated HR image $\hat{H}$. The HR training residual image is the difference between the original HR image and the reconstructed HR image $\hat{H}$, while the LR residual image is the difference between the original LR image and the downsampled version of the reconstructed HR image.

With the two training pair databases, three approaches are proposed for high frequency compensation.

### 3.1. Proposed Method 1

The first approach is shown in Figure 3. We first use the conventional interpolation method to obtain an HR image and calculate the LR residual image between the input LR image and the downsampled reconstructed HR image. Then we reconstruct the HR residual image from the LR residual image using training residual image pairs. Finally we merge the HR residual and the interpolated HR images.

### 3.2. Proposed Method 2

**Figure 4:**
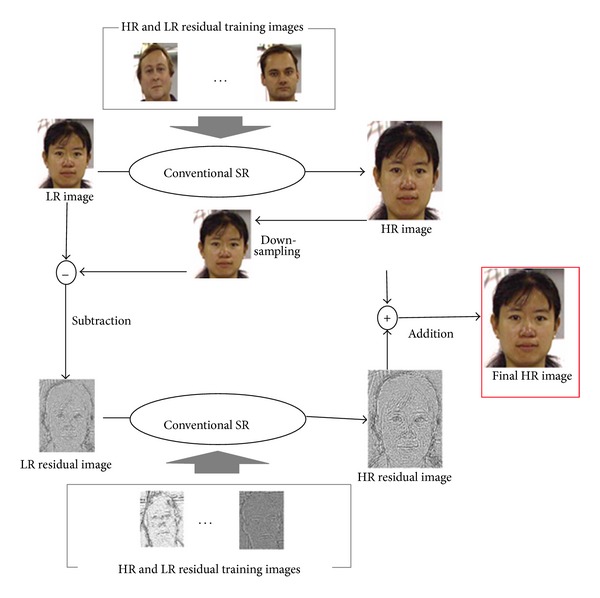
Proposed super resolution method 2.

The second approach is shown in Figure 4. We first use the conventional face hallucination method to obtain an HR image and calculate the LR residual image between the input LR image and the downsampled reconstructed HR image. Then we reconstruct the HR residual image from the LR residual image using training residual image pairs. Finally we merge the HR residual and the reconstructed HR images.

### 3.3. Proposed Method 3

**Figure 5:**
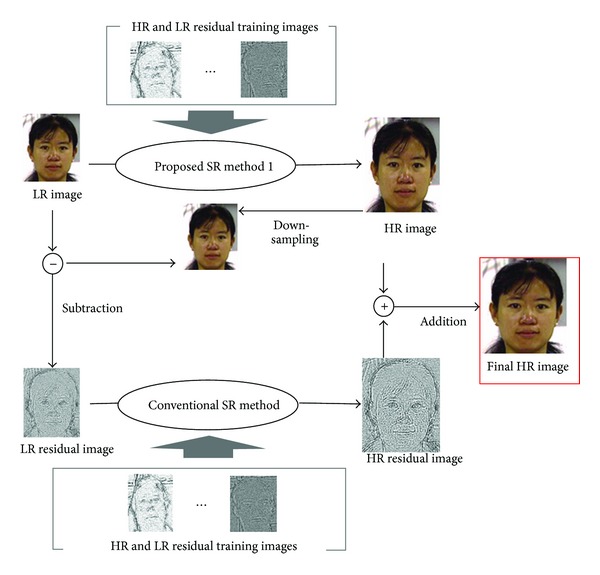
Proposed super resolution method 3.

The third approach is shown in Figure 5. We first use our proposed SR method 1 to obtain an HR image and calculate the LR residual image between the input LR image and the downsampled reconstructed HR image. Then we reconstruct the HR residual image from the LR residual image using training residual image pairs. Finally we merge the HR residual and the reconstructed HR images.

## 4. Alignment-Free Patch-Based Face Hallucination

**Figure 6:**
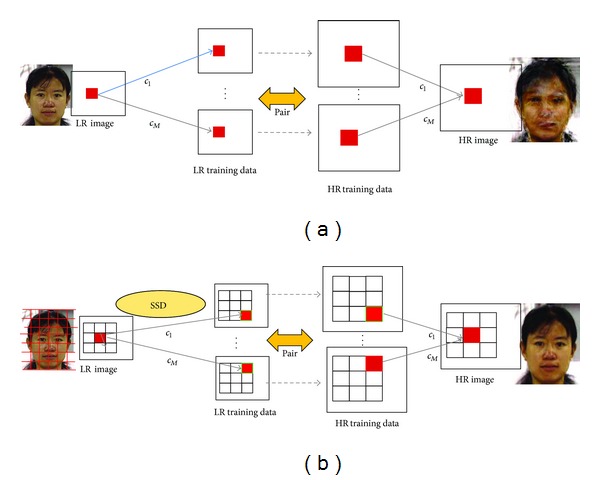
(a) Conventional face hallucination, (b) our proposed alignment-free patch-based face hallucination.

In the conventional face hallucination (Figure 6(a)), whole face image is used in training and SR reconstruction. Each pixel is reconstructed by the use of the corresponding pixel pairs at the same position. So the conventional face hallucination needs an accurate alignment of facial images in order to obtain good reconstructed HR images. With some imperfectly aligned facial datasets, it is difficult to reconstruct sharp HR images using face hallucination. In this paper, we propose a patch-based face hallucination. The basic idea of our proposed method is shown in Figure 6(b). In our proposed alignment-free patch-based face hallucination, we first segment facial images into overlapping patches and construct training patch pairs. For reconstruction of HR image, each overlapping patch in the input LR image is used as a template and its corresponding patches in LR training images are found by the use of an SSD (sum of squared difference) based template matching. Then its HR patches can be obtained by face hallucination. The whole HR image can be reconstructed by combining all of the HR patches.

## 5. Experimental Results

In order to validate the effectiveness of our proposed methods, we apply our proposed methods to two face databases. The first one is our developed MaVIC database (multiangle View, illumination and cosmetic facial image database) [13], which contains 99 aligned images of different persons and the size of each image is 320 × 400. The second one is C&P database provided by Kanade et al. [14] and Pie [11], which contains 165 imperfectly aligned frontal face images, and each image size is 264 × 320. We first generate the LR and HR image pairs by downsampling the original images. The size of LR images is 50 × 61, while the size of HR images is 200 × 244. The leave-one-out method is used in our experiments. In each database, we select one LR image randomly as a test image and its HR image is used as a ground truth image for quantitative evaluation. Other image pairs are used for training. Our proposed three methods are used for HR reconstruction of the LR test image. In order to make a comparison, the conventional face hallucination method and the bicubic interpolation method are also used for reconstructions. For each method, a total of 20 experiments with a different test image are performed. The peak signal-to-noise ratio (PSNR) [dB] is used as a quantitative measure for evaluation of the HR reconstruction performance. For C&P's imperfectly aligned facial datasets, our proposed patch face hallucination method is used with a patch size of 3 × 3 and a 1 × 1 patch that overlaps with adjacent patches.

**Figure 7:**
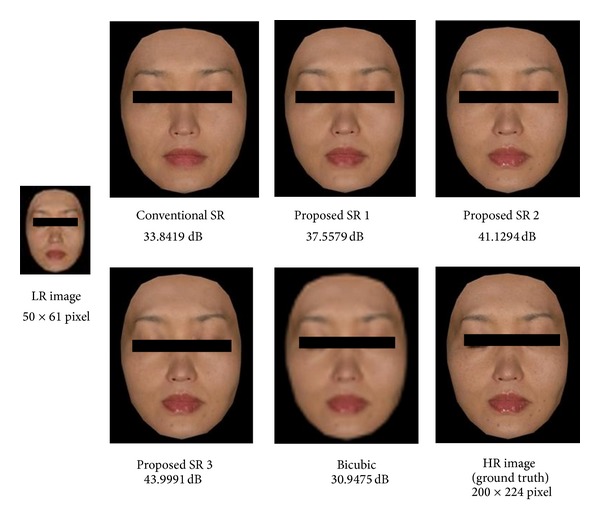
Typical reconstructed images for an aligned face image database (MaVIC).

**Figure 8:**
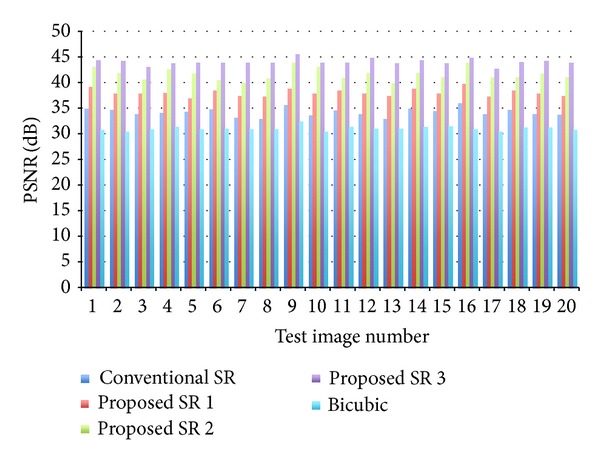
PNSR of 20 test images (MaVIC) by different methods.

Firstly, we show experimental results with the aligned face database MaVIC. A typical example is shown in Figure 7. The test LR image, which is not included in the training samples, is shown in the upper left of Figure 7. The grand truth HR image is shown in the lower right of Figure 7. Others are reconstructed HR images with their PSNR by different methods. It can be seen that the reconstructed high-resolution images obtained using our proposed approaches are much better than those obtained by conventional face hallucination method and bicubic interpolation method and the proposed method 3 shows the best performance among three proposed methods. Similar results have also been obtained with other test images. PSNR evaluation results for all test images are shown in Figure 8. The image shown in Figure 7 is corresponding to image no. 20. The mean and standard deviation over 20 experiments for each method are summarized in Table 1. It can be seen that the averaged PSNR with bicubic interpolation is about 31.16. The averaged PNSR can be improved to 34.35 [dB] by the use of conventional face hallucination. Significant improvements can be achieved by our proposed high-frequency compensation methods. The proposed method 3 shows the best performance among three proposed methods. The averaged PSNR is improved to 44.17 [dB].

**Figure 9:**
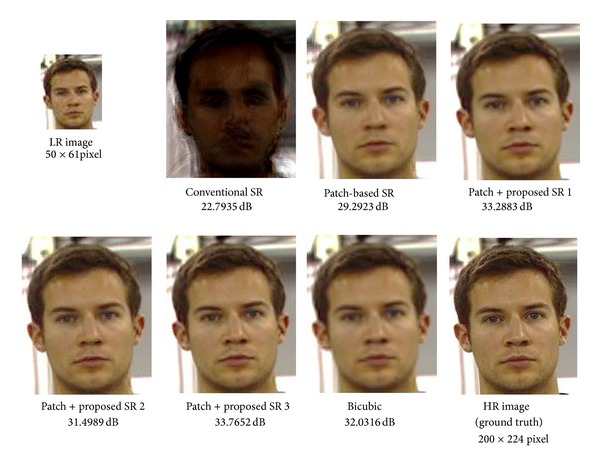
Typical reconstructed images for a nonaligned face image database (C&P).

**Figure 10:**
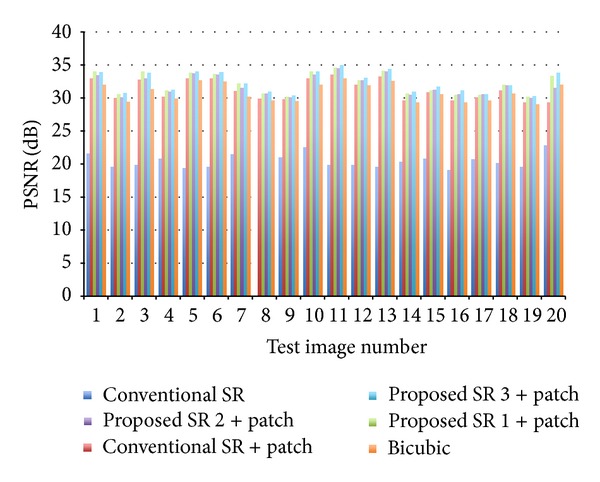
PNSR of 20 test images (C&P) by different methods.

Next, we show experimental results with the unaligned face database (C&P). Unlike experiments with MaVIC, our proposed patch-based method is used for reconstruction of HR images. A typical example is shown in Figure 9. The test LR image, which is not included in the training samples, is shown in the upper left of Figure 9. The grand truth HR image is shown in the lower right of Figure 9. Others are reconstructed HR images with their PSNR by different methods. PSNR evaluation results for all test images are shown in Figure 10. The image shown in Figure 9 is corresponding to image no. 20. The mean and standard deviation over 20 experiments for each method are summarized in Table 2. As we discussed in previous section, conventional face hallucination needs an accurate alignment of facial images in order to obtain good reconstructed HR images. For the unaligned face database C&P (usually existing face databases are not aligned), the conventional SR method, which uses whole face image for super resolution, gives a very poor result, while if we use our proposed patch-based framework, we can significantly improve the reconstructed HR image even with the conventional face hallucination method. The averaged PSNR can be improved from 20.42 [dB] to 31.21 [dB]. It means that our proposed method is an alignment-free SR method. Furthermore, the HR image with higher quality can be achieved by our proposed high-frequency compensation methods. As well as the results with MaVIC, the proposed method 3 shows the best performance among three proposed methods. The averaged PSNR is improved to 32.96 [dB]. Though smaller patch size may improve the quality of reconstructed HR images, it will increase the computation cost. Optimum patch size depends on the purpose and applications.

## 6. Conclusions

We proposed a residual image compensation framework together with a patch-based alignment-free method to improve the reconstruction quality for face hallucination. The basic idea of our proposed residual image compensation framework was to reconstruct or estimate a residual image, which can be used to compensate the high-frequency components of the reconstructed high-resolution image. Three approaches based on our proposed framework were proposed. In the patch-based alignment-free face hallucination, we first segmented facial images into overlapping patches and constructed training patch pairs. For an input LR image, the overlapping patches are also used to obtain the corresponding HR patches by face hallucination. The whole HR image can then be reconstructed by combining all of the HR patches. The effectiveness of our proposed methods has been demonstrated on both the aligned face database (MaVIC) and the unaligned face database (C&P). The reconstructed high-resolution images obtained using our proposed approaches are much better than those obtained by conventional face hallucination method and bicubic interpolation method. The averaged PSNR of reconstructed HR images was improved from 34.35 [db] to 44.17 [dB] for the aligned face database (MaVIC) and from 20.42 [dB] to 32.40 [dB] for the unaligned face database (C&P). The proposed high-frequency compensation method 3 shows the best performance among three proposed approaches.

## Figures and Tables

**Figure 1:**
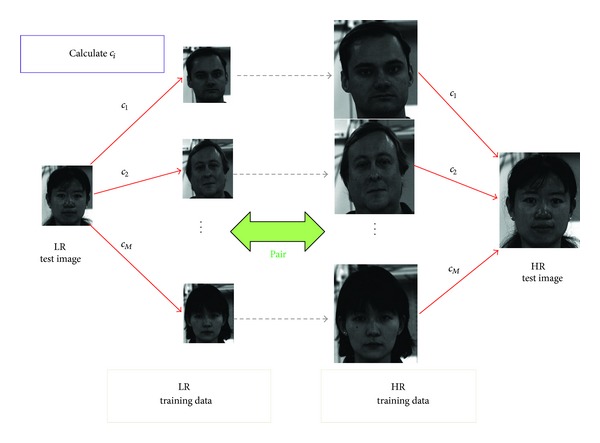
Schematic diagram of face hallucination.

**Table 1:** Comparison of the averaged PSNR for an aligned face database (MaVIC).

Method	Conventional SR [10]	Proposed SR 1	Proposed SR 2	Proposed SR 3	Bicubic
Mean	34.35	38.18	41.74	44.17	31.16
Stv.	0.81	0.72	1.18	0.60	0.45

**Table 2:** Comparison of the averaged PSNR for a nonaligned face image database (C&P).

Methods	Conventional SR [10]	Conventional SR + patch	Proposed SR 1 + patch	Proposed SR 2 + patch	Proposed SR 3 + patch	Bicubic
Mean	20.42	31.21	32.49	32.19	32.96	30.86
Stv.	1.03	1.54	1.60	1.50	1.58	1.37
